# A Systematic Study on DNA Barcoding of Medicinally Important Genus *Epimedium* L. (Berberidaceae)

**DOI:** 10.3390/genes9120637

**Published:** 2018-12-17

**Authors:** Mengyue Guo, Yanqin Xu, Li Ren, Shunzhi He, Xiaohui Pang

**Affiliations:** 1 Key Lab of Chinese Medicine Resources Conservation, State Administration of Traditional Chinese Medicine of the People’s Republic of China, Institute of Medicinal Plant Development, Chinese Academy of Medical Sciences & Peking Union Medical College, Beijing 100193, China; guomy0908@hotmail.com (M.G.); renliwangyi@hotmail.com (L.R.); 2 College of Pharmacy, Jiangxi University of Traditional Chinese Medicine, Nanchang 330004, China; 20081016@jxutcm.edu.cn; 3 Department of Pharmacy, Guiyang College of Traditional Chinese Medicine, Guiyang 550002, China; shunzhihe2018@hotmail.com

**Keywords:** *Epimedium*, DNA barcode, identification, super barcode

## Abstract

Genus *Epimedium* consists of approximately 50 species in China, and more than half of them possess medicinal properties. The high similarity of species’ morphological characteristics complicates the identification accuracy, leading to potential risks in herbal efficacy and medical safety. In this study, we tested the applicability of four single loci, namely, *rbcL*, *psbA*-*trnH*, internal transcribed spacer (ITS), and ITS2, and their combinations as DNA barcodes to identify 37 *Epimedium* species on the basis of the analyses, including the success rates of PCR amplifications and sequencing, specific genetic divergence, distance-based method, and character-based method. Among them, character-based method showed the best applicability for identifying *Epimedium* species. As for the DNA barcodes, *psbA*-*trnH* showed the best performance among the four single loci with nine species being correctly differentiated. Moreover, *psbA*-*trnH* + ITS and *psbA*-*trnH* + ITS + *rbcL* exhibited the highest identification ability among all the multilocus combinations, and 17 species, of which 12 are medicinally used, could be efficiently discriminated. The DNA barcode data set developed in our study contributes valuable information to Chinese resources of *Epimedium*. It provides a new means for discrimination of the species within this medicinally important genus, thus guaranteeing correct and safe usage of *Herba Epimedii*.

## 1. Introduction

Over 200 million people suffer from osteoporosis around the world, and the prevalence of osteoporosis keeps rising with the increasing elderly population [1]. In 2005, the total fractures were more than 2 million, costing nearly $17 billion in the United States. The rapid growth in the disease burden was also projected from 2005 to 2025 [2]. For a long time, Chinese herbal medicine has been used to treat fractures and joint diseases in China. *Herba Epimedii* (Yinyanghuo) is one of the most widely used herbs that are prescribed in formulas to treat osteoporosis [3], and its extract can reduce the occurrence of osteoporosis both in experimental models and clinical studies [4]. The term Yinyanghuo was first listed in Shen Nong Ben Cao Jing as a middle-grade herb during 200–300 B.C., and many species in genus *Epimedium* L. (Berberidaceae) have been used in traditional Chinese medicine (TCM) for a long time to nourish the kidney and reinforce the Yang [5,6]. At present, five species are recorded in the Chinese pharmacopoeia. The dried leaves of *Epimedium brevicomu* Maxim, *Epimedium sagittatum* (Sieb. et Zucc) Maxim, *Epimedium pubescens* Maxim, and *Epimedium koreanum* Nakai are the sources of Epimedii Folium (Yinyanghuo), and the dried leaves of *E. wushanense* T.S. Ying are the resources of Epimedii Wushanensis Folium (Wushan Yinyanghuo) [7]. Simultaneously, *Epimedium* species are used medically in other Asian countries. For instance, *E*. *sagittatum* and *E*. *grandiflorum* have been used to treat impotence, prospermia, hyperdiuresis, and osteoporosis in Japan [6], and *E. koreanum* has been used in Korea as an aphrodisiac and hypotensive [8]. Up to now, more than 270 compounds were isolated from *Epimedium* species. The major active components are flavonoids and corresponding derivatives, with antitumor, antioxidative, antiosteoporosis, and protective effects [9,10,11].

In terms of the great medicinal value of *Epimedium* species, considerable scientific interest has been aroused in this genus. The completely accurate identification of the plant material is the basis for a scientific study. However, the classification and discrimination of *Epimedium* species have always been controversial. Genus *Epimedium* consists of approximately 58 species distributed from Japan in Asia to Algeria in North Africa [12]. Most of the *Epimedium* species exist in Eastern Asia and Mediterranean countries [12], and approximately 50 species have been reported in China [13,14,15]. The first *Epimedium* species, *E. alpinum* L., was recorded by Linnaeus whereas the most comprehensive classification system of this genus was established by Stearn [12]. In Stearn’s monograph, genus *Epimedium* is divided into subgenera *Rhizophyllum* and *Epimedium* on the basis of the flower and leaf morphology, C-banding of the chromosomes [16], and geographical distribution [12]. Furthermore, subgenus *Epimedium* is divided into four sections, namely, *Epimedium*, *Polyphyllon*, *Macroceras*, and *Diphyllon*. According to the corolla characteristics, section *Diphyllon*, which has approximately 47 known species in China, is further subdivided into four series, namely, *Campanulatae* Stearn, *Davidianae* Stearn, *Dolichocerae* Stearn, and *Brachycerae* Stearn. Chinese sect. *Diphyllon* reaches the highest species diversity with more than 80% species in *Epimedium* [17], presenting numerous taxonomic controversies [18,19,20,21]. Morphological classification experts identify and publish new species according to their leaf and corolla characteristics [12]. Most species in this genus have similar leaf morphology (three branches from the stem and three leaves in every branch). Thus, the species identification on the basis of leaf morphology becomes difficult when the short flowering time has passed. For instance, *E*. *accuminata* and *E*. *pubescens* are difficult to differentiate due to their least variable leaf morphology [12]. Meanwhile, because of the similar shape among different species and intraspecific variation caused by geographical distribution, distinguishing closely related species becomes difficult. For instance, *E*. *sagittatum* and its related species, namely, *E*. *sagittatum* complex, present the most disputable questions in taxonomy due to the high morphological variations with extensive geographical distribution [20,22]. These taxonomic issues mentioned above complicate the species discrimination in this genus. At present, approximately 23 *Epimedium* species not recorded in the Chinese pharmacopeia, such as *E. acuminatum*, *E. miryanthum*, and *E. leptorrhizum*, are commonly used as Yinyanghuo in particular localities of Guizhou, Sichuan, Chongqing, Jiangxi, Hunan, and Hubei provinces in China [23,24]. Considering differences in bioactive constituents among *Epimedium* species [6], incorrect identification of species is likely to result in potential risks in herbal efficacy and medical safety. Furthermore, *Herba Epimedii* is an herb that is harvested in the wild and is on the edge of extinction [25]. Improper or wrong harvest may induce the reduction of species abundance in genus *Epimedium*. Thus, effective methods for the accurate discrimination and sustainable utilization of the *Epimedium* resources are urgently needed. 

In order to resolve the identification problem, some researchers have attempted to use molecular methods to discriminate *Epimedium* species. For example, 5S ribosomal RNA (rRNA) gene spacer sequencing [26], expressed sequence tag (EST) dataset [27], and microsatellites [28,29,30] methods have been applied to identify medicinal *Epimedium* species. Some identification problems of *Epimedium* species were settled in these studies. However, a comprehensive discrimination study of medicinally important *Epimedium* species is absent at present. Recently, DNA barcoding technique has been widely used for species discrimination. Up to now, *matK*, *rbcL*, *psbA*-*trnH*, internal transcribed spacer (ITS), and ITS2 are the most commonly used regions for DNA barcoding in plants [31,32,33,34]. In this study, we systematically evaluated the feasibility and identification efficiency of four generally acknowledged single loci, namely, *rbcL*, *psbA*-*trnH*, ITS, and ITS2, and their combinations to discriminate the *Epimedium* species distributed in China to identify the most suitable barcodes for genus *Epimedium* and provide effective information for the species identification of this genus. 

## 2. Materials and Methods

### 2.1. Plant Materials

A total of 72 samples representing 37 *Epimedium* species, of which 20 are medicinally used, were collected from the Jiangxi, Guizhou, Hubei, and Jilin provinces in China. In addition to the widely distributed *Epimedium* species, some narrowly distributed species of the genus in China were collected as well. For instance, *E*. *dewuense* and *E*. *shuichengense* are endemic species to certain areas of Guizhou province. *E*. *zhushanense* only grows in Zhushan County in the Hubei province, and *E*. *tianmenshanensis* is merely distributed in Hunan province. Moreover, some rare species, including *E*. *pauciflorum*, *E*. *platypetalum*, *E*. *lishihchenii*, *E*. *franchetii*, *E*. *glandulosopilosum*, *E*. *truncatum*, *E*. *dolichostemon*, *E*. *pudingense*, *E*. *ilicifolium*, *E*. *borealiguizhouense*, and *E*. *qingchengshanense*, are mostly distributed in certain areas with extremely limited resources and survive with difficulty when transplanted. All samples were authenticated by Prof. Yanqin Xu of Jiangxi University of Traditional Chinese Medicine and Prof. Shunzhi He of Guiyang College of Traditional Chinese Medicine. The details of the samples are listed in Table S1. All corresponding voucher samples were deposited in the Herbarium of the Institute of Medicinal Plant Development, Chinese Academy of Medicinal Sciences & Peking Union Medical College, Beijing, China.

### 2.2. DNA Isolation, Amplification, and Sequencing

Approximately 30 mg fresh leaf from each sample was ground for 2 min at a frequency of 30 times/s in a FastPrep bead mill (Retsch MM400, Haan, Germany). The total genomic DNA was extracted using the Universal Plant Genome DNA Kit (Tiangen Biotech, Beijing, China) according to the manufacturer’s instructions. Short fragments of the specific regions of the plastid (*rbcL*), noncoding (*psbA*-*trnH*), and nuclear DNA (ITS, including 18S, ITS1, 5.8 S, ITS2, and 28 S) sequences were amplified from the extracts. The ITS2 sequence was retrieved from the ITS region directly. Universal primers for candidate barcodes and reaction conditions were used as previously reported [35,36,37]. The primers and reaction conditions used to amplify these regions are listed in Table S2. PCR was performed in 25 µL of the reaction system, containing 2 µL of template DNA (approximately 30 ng), 12.5 µL of 2× Taq PCR Master Mix (Aidlab Biotechnologies Co., Ltd., Beijing, China), and 1 µL of each primer (2.5 µmol/L), and filled with double-distilled water. The purified PCR products were sequenced in both directions by using an ABI 3730XL sequencer (Applied Biosystems, Inc., Foster City, CA, USA) on the basis of the Sanger sequencing method by the Major Engineering laboratory of the Chinese Academy of Agricultural Sciences (Beijing, China). Altogether, 70 samples representing 36 species were sequenced successfully with all the three loci (*rbcL*, *psbA*-*trnH*, and ITS), and 210 new sequences were submitted to GenBank under the accession number MG837275-MG837475 and MH252065-MH252073 (Table S1). These sequences were used in the subsequent analysis.

### 2.3. Statistical Analysis

Consensus sequences and contig generation were performed using the CodonCode Aligner V 7.1.1 (CodonCode Co., Dedham, MA, USA). The ITS sequences were subjected to hidden Markov model analysis to obtain the complete ITS2 sequences [38]. Sequences from each DNA region were aligned using MEGA 6.0 [39]. For species with more than three individuals, the average intraspecific distance, theta, and coalescent depth were calculated to evaluate the intraspecific variation based on the Kimura two-parameter (K2P) model; the average interspecific distance, minimum interspecific distance, and theta primer were used to assess the interspecific divergence using the K2P model [40,41,42]. To assess the potential of each single locus and their combinations for accurate species discrimination, distance-based methods (TaxonDNA and neighbor-joining trees) and character-based approach (BLOG) were employed for species with more than one individual. The best match (BM) and best close match (BCM) tests in the Species Identifier 1.8 program of TaxonDNA were run [43]. The neighbor-joining (NJ) trees of each single locus and their combinations were constructed using MEGA 6.0, and bootstrap tests were performed with 1000 replicates [39]. The character-based DNA barcode method in BLOG (Barcoding with LOGic) 2.0 was applied to classify species with more than one individual [44]. Each single locus and their combinations were subjected to 100% slicing within species level with a maximum of 500 iterations (GRASPITER = 500) and a maximum time of 5 min for analysis (GRASPSECS = 300). The logic formula with the lowest false positive rate against the reference dataset was taken as discrimination basis. Moreover, species that could be identified were shown using Venn diagrams. 

## 3. Results

### 3.1. Efficiency of PCR Amplification and Sequencing

The three loci, *rbcL*, ITS, and *psbA*-*trnH*, all showed high PCR amplification and sequencing efficiency (100%). Moreover, the effective sequence ratio of *rbcL* and *psbA*-*trnH* were the same (100%), followed by ITS (97.2%). Detailed information regarding the PCR amplification and sequencing efficiency of the candidate barcodes is provided in Table S1. 

### 3.2. Genetic Divergence Determination 

The length of the aligned sequence (base pairs) and variable sites for the *rbcL*, *psbA*-*trnH*, ITS, and ITS2 regions were 703/10, 572/59, 705/45, and 247/23, respectively. Six parameters were used to characterize inter- and intraspecific divergence. Results indicated that *psbA*-*trnH* had the highest interspecific divergence, followed by ITS2 and ITS. Meanwhile, *rbcL* exhibited a relatively lower interspecific divergence compared with the other regions (Table S3). At the intraspecific level, *rbcL* showed the lowest divergence, while *psbA*-*trnH* displayed the highest variation level (Table S3). 

### 3.3. Evaluation of the Identification Efficiency of the DNA Barcodes

**Figure 1:**
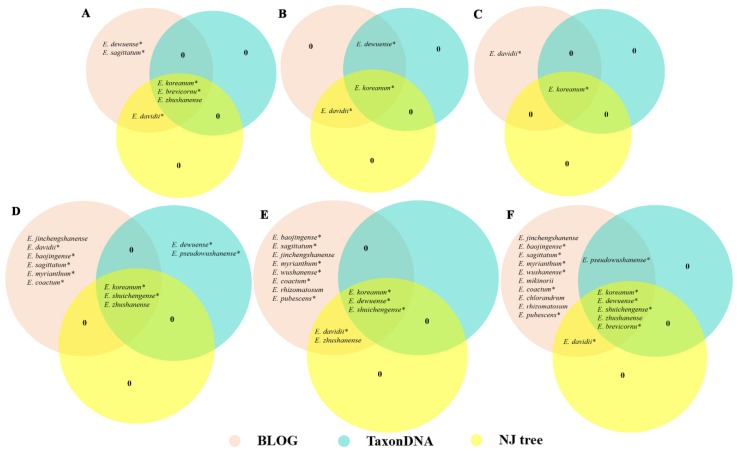
Species that can be identified based on three methods for each single locus and their combinations by using Venn diagrams. A, ITS; B, ITS2; C, *rbcL*; D, *psbA*-*trnH*; E, *psbA*-*trnH* + ITS2/*psbA*-*trnH* + ITS2 + *rbcL*; F, *psbA*-*trnH* + ITS/*psbA*-*trnH* + ITS + *rbcL.* “*” represents medicinally used species.

In the present study, the distance-based method, namely, TaxonDNA, was used to assess the applicability of the different regions for species discrimination. Similar identification efficiency was obtained based on the BM and BCM methods (Table 1). For the single locus, *psbA*-*trnH* exhibited the highest species identification efficiency (29.62%), followed by the ITS (22.22%) and ITS2 regions (18.51%). Meanwhile, the *rbcL* region showed the lowest resolution rate of 3.70%. In the two loci combinations, *psbA*-*trnH* + ITS2 showed a higher resolution rate (38.88%) than *psbA*-*trnH* + ITS (37.03%). Moreover, compared with the two loci combinations, the resolution rate was not increased when three loci (*psbA*-*trnH* + ITS + *rbcL* and *psbA*-*trnH* + ITS2 + *rbcL*) were combined (37.03%). Results showed that only six species (*E*. *koreanum*, *E*. *dewuense*, *E*. *zhushanense*, *E*. *shuichengense*, *E. brevicornu*, and *E*. *pseudowushanense*) could be identified efficiently using single locus and their combinations (Figure 1). 

Second only to ITS region, *psbA*-*trnH* exhibited comparatively higher identification efficiency among the four single loci by using the NJ tree method (Figure 1). Three species, namely, *E*. *koreanum*, *E*. *zhushanense*, and *E*. *shuichengense*, could be identified using the locus *psbA*-*trnH*. Moreover, results suggested that the number of species that can be authenticated was increased when two or three loci were combined (Figure 1). Six species, namely, *E*. *koreanum*, *E*. *dewuense*, *E*. *zhushanense*, *E*. *shuichengense*, *E*. *davidii*, and *E. brevicornu*, could be discriminated by *psbA*-*trnH* + ITS and *psbA*-*trnH* + ITS + *rbcL*. Additionally, five *Epimedium* species, namely, *E*. *koreanum*, *E*. *dewuense*, *E*. *zhushanense*, *E*. *shuichengense*, and *E*. *davidii*, could be differentiated by *psbA*-*trnH* + ITS2 and *psbA*-*trnH* + ITS2 + *rbcL*. Detailed information regarding the NJ trees of each single locus and their combinations is provided in Figures S1 and S2. 

Compared with the distance-based method, the identification efficiency of each single locus and their combinations was significantly improved by using the character-based approach (Table 1). Among the four single loci, *psbA*-*trnH* displayed the highest resolution rate (61.11%) with nine species being identified. By contrast, *rbcL* showed the lowest (14.81%). Moreover, combination of barcodes increased the species identification efficiency significantly. Among the four multilocus combinations, *psbA*-*trnH* + ITS and *psbA*-*trnH* + ITS + *rbcL* both showed the highest resolution rate (92.59%). Aside from *E. acuminatum*, *E. leptorrhizum*, and *E. epsteinii*, a total of 17 species, of which 12 are medicinally used, could be distinguished (Figure 1). The logic formulas to identify the 17 species by using *psbA*-*trnH* + ITS region are listed in Table 2.

## 4. Discussion

### 4.1. Development of DNA Barcode Resources for Genus Epimedium 

In our research, four universal candidate barcodes, namely, *rbcL*, *psbA*-*trnH*, ITS, and ITS2, were assessed for their applicability in identifying the *Epimedium* species. All three regions (*rbcL*, *psbA*-*trnH*, and ITS) showed high rates of sequencing recovery. The *psbA*-*trnH* intergenic spacer was reported as one of the most variable plastid regions in the angiosperms [35]. In our study, it demonstrated the highest interspecific divergence between species of *Epimedium*. Meanwhile, it exhibited better identification capability than the other regions for *Epimedium* species based on both distance (TaxonDNA and NJ tree) and character (BLOG) analyses. The BLOG method displayed the highest identification ability with nine species being differentiated, whereas five and three species can be respectively discriminated by TaxonDNA and NJ tree. Thus, *psbA*-*trnH* region may be used as a potential barcode to discriminate the *Epimedium* species. Second only to *psbA*-*trnH*, ITS region showed good identification ability with six species being differentiated. The China Plant BOL Group suggested that ITS/ITS2 should be incorporated into the core barcode for seed plants [45]. Here, ITS2 showed lower identification efficiency than ITS based on both distance- and character-based methods, which could be attributed to lower interspecific divergence of ITS2 for *Epimedium* species.

Considering the limited discriminatory power of the single-locus barcode, a multigene tiered approach for barcoding plants was recommended by Newmaster et al. [46]. In this study, the combination of two and three loci showed better identification efficiency than that of the single locus. The combination of two loci *psbA*-*trnH* and ITS, as well as the combination of three loci *psbA*-*trnH*, ITS, and *rbcL*, showed the highest discrimination power among all the multilocus combinations. As for the analytical methods, the character-based method exhibited the highest identification efficiency with 17 species being discriminated. The character-based method (BLOG), suggested to be efficient and precise, could provide diagnostic formulas listing the species-specific nucleotides to differentiate species from others [47]. On the contrary, the distance-based method showed lower resolution rate than the character-based method, and only six species could be discriminated. The intraspecific and interspecific genetic distances overlapped in our study (Table S3), making authentication of these species difficult by using BM test, BCM test, and NJ trees. Therefore, the character-based method is a more appropriate choice to identify *Epimedium* species. 

This study contributes with DNA barcodes for more than 70% of the species in *Epimedium*, covering nearly all the commonly used medicinal plant species and rare species. For each species, information of the four universal barcodes, namely, *rbcL*, *psbA*-*trnH*, ITS, and ITS2, were provided. The dataset developed could provide assistance for accurate species identification, sustainable recourse utilization, and new medicinal resources development of this genus.

### 4.2. Chloroplast Genome-Based Super Barcode Has the Potential to Solve the Problem of Species Discrimination and Classification in Genus Epimedium

A number of taxonomic controversies exist in the Chinese sect. *Diphyllon* [18,19,48], and efficient methods for species identification and classification are lacking. In our study, the commonly used DNA barcodes were effective in identifying 17 *Epimedium* species, but the phylogenetic relationships of Chinese sect. *Diphyllon* were poorly resolved. The unresolved phylogeny of genus *Epimedium* might be ascribed to the following taxonomic issues. First, some species published earlier were only based on one or two specimens or even fragmentary specimens, resulting in the inaccurate description of the morphological characteristics. *Epimedium baojingensis* and *E. zhushanense* were believed to be the only species with unifoliolate leaves when they were published. However, the investigation of Zhang et al. [48] revealed that leaves of these species are usually trifoliolate and occasionally unifoliolate. Second, using the small variations as the reference to publish new species tends to ignore the possible connection between the new species and model specimens, which may lead to unnecessary publication to some extent and even cause problems for further study. Third, incomplete breeding isolation between the species and existence of natural hybrids may also complicate the relationships of the Chinese *Epimedium* species, thereby increasing the difficulty of morphological identification and taxonomy research [12]. Thus, a more effective method is needed to further research the discrimination and classification of this genus. Recently, the chloroplast (cp) genomes of plants have been applied as a useful tool for phylogenetic studies and species identification [49,50,51]. The cp genome can significantly increase the resolution at low taxonomic levels in plant phylogeny; it was proposed as a species-level DNA barcode [52] and has been used as a plant barcode to discriminate closely related species [52,53,54]. The complete cp genome with more variation information than single or multiple DNA barcodes was also suggested as a super barcode [55], which may be a solution for the identification and classification problems in the *Epimedium* species. Zhang et al. [13] sequenced the complete cp genomes of five *Epimedium* species and found that the phylogenetic relationships among these cp genomes were consistent with the updated system. However, the cp genome study of 90% of the approximately 50 *Epimedium* species distributed in China still has not been conducted. Additionally, only one sample was sequenced for each species, which was not adequate to demonstrate the evolutionary relationships and divisions within the section *Diphyllon* because some *Epimedium* species have large intraspecific variations. Therefore, more systematic and in-depth classification and identification investigation on the basis of the complete cp genomes of a large sample size are urgently needed. Thus, the cp genome-based super barcode will be utilized to study the identification and evolution relationships of all Chinese *Epimedium* species in our further research. Further research is expected to solve the complex problems in taxonomy and species discrimination and to guarantee the medical safety of species in genus *Epimedium*.

## Figures and Tables

**Table 1:** Identification efficiency of the four loci on the basis of the BM, BCM, and BLOG analytical methods.

	Best Match			Best Close Match (Threshold 3%)				BLOG		
Region	Correct	Ambiguous	Incorrect	Correct	Ambiguous	Incorrect	No Match	Correct	Not Classified	Wrong
*psbA-trnH*	29.62%	27.77%	42.59%	29.62%	27.77%	38.88%	3.70%	61.11%	38.89%	0.00%
ITS	22.22%	70.37%	7.40%	22.22%	70.37%	7.40%	0.00%	48.15%	51.85%	0.00%
ITS2	18.51%	74.07%	7.40%	18.51%	74.07%	7.40%	0.00%	24.07%	75.93%	0.00%
*rbcL*	3.70%	96.29%	0.00%	3.70%	96.29%	0.00%	0.00%	14.81%	85.19%	0.00%
*psbA-trnH* + ITS	37.03%	24.07%	38.88%	37.03%	24.07%	38.88%	0.00%	92.59%	7.41%	0.00%
*psbA-trnH* + ITS2	38.88%	25.92%	35.18%	38.88%	25.92%	33.33%	3.70%	77.78%	22.22%	0.00%
*psbA-trnH* + ITS + *rbcL*	37.03%	18.51%	44.44%	37.03%	18.51%	44.44%	0.00%	92.59%	7.41%	0.00%
*psbA-trnH* + ITS2 + *rbcL*	37.03%	24.07%	38.88%	37.03%	24.07%	38.88%	0.00%	81.48%	18.52%	0.00%

ITS, internal transcribed spacer; BLOG, Barcoding with LOGic.

**Table 2:** Diagnostic formulas of *psbA-trnH* + ITS region generated by BLOG for species identification. “*” represents medicinally used species.

Species	Formulas	Score	Coverage
E. davidii *	pos1043 = T	0.136	1
E. baojingense *	pos314 = G OR pos1085 = C	0.174	1
E. koreanum *	pos201 = T	0.136	1
E. dewuense *	pos395 = C & pos1220 = G	0.136	1
E. sagittatum *	pos201 = G & pos320 = T & pos395 = T & pos411 = G & pos1052 = T	0.24	1
	OR pos91 = C		
	OR pos113 = A		
*E. jinchengshanense*	pos224 = C & pos411 = T & pos1220 = G	0.136	1
	OR pos411 = G & pos1052 = A		
*E. chlorandrum*	pos90 = T & pos150 = G & pos201 = G & pos224 = A & pos395 = T & pos411 = T & pos536 = A & pos1043 = C & pos1052 = T & pos1220 = A & pos1252 = G	0.174	1
	OR pos1026 = C & pos1182 = T		
E. pseudowushanense *	pos90 = T & pos91 = A & pos224 = C & pos411 = T & pos788 = C & pos1182 = C & pos1220 = A	0.136	1
*E. rhizomatosum*	pos411 = T & pos1052 = A	0.136	1
E. pubescens *	pos90 = T & pos150 = G & pos201! = G & pos201! = T & pos395 = T	0.174	1
	OR pos90 = G & pos224 = C & pos411 = T & pos1026 = C & pos1182 = C		
	OR pos320 = A		
E. shuichengense *	pos201 = A & pos1052 = T	0.136	1
E. myrianthum *	pos90 = G & pos411 = G	0.208	1
	OR pos113 = G & pos150 = A & pos314 = A & pos730 = A		
E. wushanense *	pos164 = T & pos224 = A & pos411 = T & pos1085 = T & pos1220 = G	0.174	1
	OR pos90 = G & pos201 = G & pos224 = A & pos411 = T & pos1085 = T		
E. brevicornu *	pos1252 = C	0.136	1
E. coactum *	pos505 = C & pos536 = C	0.136	1
	OR pos1026 = A		
*E. mikinorii*	pos788 = T	0.136	1
	OR pos90 = T & pos224 = C & pos411 = G & pos1220 = A		
*E. zhushanense*	pos164 = G	0.136	1

ITS, internal transcribed spacer; BLOG, Barcoding with LOGic.

## Supplementary Materials

The following are available online at http://www.mdpi.com/2073-4425/9/12/637/s1, **Figure S1**. NJ trees of *Epimedium* species on the basis of the *psbA*-*trnH* (A), *rbcL* (B), ITS (C), and ITS2 (D) regions. The bootstrap scores (1000 replicates) are shown (≥50%) for each branch. Figure S2. NJ trees of *Epimedium* species on the basis of four multilocus combinations. The bootstrap scores (1000 replicates) are shown (≥50%) for each branch. A, *psbA*-*trnH* + ITS; B, *psbA*-*trnH* + ITS2; C, *psbA*-*trnH* + ITS2 + *rbcL*; D, *psbA*-*trnH* + ITS + *rbcL*. Table S1. Voucher information, PCR amplification and sequencing efficiency of the candidate barcodes, and GenBank accession numbers for *Epimedium* plant samples in this study. Table S2. List of universal primers and reaction conditions for candidate barcodes used in this study. Table S3. Inter- and intraspecific genetic divergences of the four loci.
