# Supplementary material for: A Systematic Study on DNA Barcoding of Medicinally Important Genus Epimedium L. (Berberidaceae)
Source: Genes (Basel). 2018 Dec 17;9(12):637. doi: 10.3390/genes9120637 (PMC6316794; doi:10.3390/genes9120637)
Supplement: Supplementary file 1 [file genes-09-00637-s001.zip › supplementary tables.docx]

**Supplementary Tables: A systematic study on DNA barcoding of medicinally important genus *Epimedium* L. (Berberidaceae)**

**Mengyue Guo ^1^, Yanqin Xu^2^, Li Ren ^1^, Shunzhi He ^3^, Xiaohui Pang ^1,^ ***

**Table S1.** Voucher information, PCR amplification and sequencing efficiency of the candidate barcodes, and GenBank accession numbers for *Epimedium* plant samples in this study.

| Species | Voucher No. | Sampling Part | Locality information | PCR amplification and sequencing success / effective sequence | | | GenBank Accession No. | | | |  |
| --- | --- | --- | --- | --- | --- | --- | --- | --- | --- | --- | --- |
|  |  |  |  | *psbA*-*trnH* | *rbcL* | ITS | | *psbA*-*trnH* | *rbcL* | ITS | |
| *Epimedium davidii** | BX01 | Leaf | Baoxing, Sichuan | + / + | + / + | + / + | | MG837342 | MG837409 | MG837275 | |
| *Epimedium davidii** | BX02 | Leaf | Yuexi, Sichuan | + / + | + / + | + / + | | MG837343 | MG837410 | MG837276 | |
| *Epimedium pseudowushanense** | NWS01 | Leaf | Leishan, Guizhou | + / + | + / + | + / + | | MG837370 | MG837437 | MG837303 | |
| *Epimedium pseudowushanense** | NWS02 | Leaf | Jianhe, Guizhou | + / + | + / + | + / + | | MG837369 | MG837436 | MG837302 | |
| *Epimedium pauciflorum* | SH01 | Leaf | Wenchuan, Sichuan | + / + | + / + | + / + | | MG837382 | MG837449 | MG837315 | |
| *Epimedium shuichengense** | SC01 | Leaf | Shuicheng, Guizhou | + / + | + / + | + / + | | MG837385 | MG837452 | MG837318 | |
| *Epimedium shuichengense** | SC02 | Leaf | Shuicheng, Guizhou | + / + | + / + | + / + | | MG837384 | MG837451 | MG837317 | |
| *Epimedium wushanense** | WS01 | Leaf | Guizhou | + / + | + / + | + / + | | MG837393 | MG837460 | MG837326 | |
| *Epimedium wushanense** | WS02 | Leaf | Xingshan, Hubei | + / + | + / + | + / + | | MG837392 | MG837459 | MG837325 | |
| *Epimedium wushanense** | WS03 | Leaf | Guizhou | + / + | + / + | + / + | | MG837394 | MG837461 | MG837327 | |
| *Epimedium mikinorii* | ZJ01 | Leaf | Leishan, Guizhou | + / + | + / + | + / + | | MG837403 | MG837470 | MG837336 | |
| *Epimedium mikinorii* | ZJ02 | Leaf | Enshi, Hubei | + / + | + / + | + / + | | MG837404 | MG837471 | MG837337 | |
| *Epimedium hunanense** | HN01 | Leaf | Hefeng, Hubei | + / + | + / + | + / + | | MH252068 | MH252071 | MH252065 | |
| *Epimedium epsteinii* | ZIJ01 | Leaf | Shaoguan, Guangdong | + / + | + / + | + / + | | MG837407 | MG837474 | MG837340 | |
| *Epimedium epsteinii* | ZIJ02 | Leaf | Jianshi, Hubei | + / + | + / + | + / + | | MG837408 | MG837475 | MG837341 | |
| *Epimedium baojingense** | BJ01 | Leaf | Guiyang, Guizhou | + / + | + / + | + / + | | MG837344 | MG837411 | MG837277 | |
| *Epimedium baojingense** | BJ02 | Leaf | Changyang, Hubei | + / + | + / + | + / + | | MG837345 | MG837412 | MG837278 | |
| *Epimedium baojingense** | BJ03 | Leaf | Yongshun, Hunan | + / + | + / + | + / + | | MG837346 | MG837413 | MG837279 | |
| *Epimedium acuminatum** | CM01 | Leaf | Guiyang, Guizhou | + / + | + / + | + / + | | MG837349 | MG837416 | MG837282 | |
| *Epimedium acuminatum** | CM02 | Leaf | Nanchuan, Chongqing | + / + | + / + | + / + | | MG837350 | MG837417 | MG837283 | |
| *Epimedium acuminatum** | CM03 | Leaf | Yaan, Sichuan | + / + | + / + | + / + | | MG837351 | MG837418 | MG837284 | |
| *Epimedium acuminatum** | CM04 | Leaf | Leshan, Sichuan | + / + | + / + | + / + | | MG837352 | MG837419 | MG837285 | |
| *Epimedium acuminatum** | CM05 | Leaf | Yaan, Sichuan | + / + | + / + | + / + | | MG837353 | MG837420 | MG837286 | |
| *Epimedium acuminatum** | CM06 | Leaf | Yanhe, Guizhou | + / + | + / + | + / + | | MH252069 | MH252072 | MH252066 | |
| *Epimedium jinchengshanense* | JCS01 | Leaf | Bazhong, Sichuan | + / + | + / + | + / + | | MG837362 | MG837429 | MG837295 | |
| *Epimedium jinchengshanense* | JCS02 | Leaf | Beichuan, Sichuan | + / + | + / + | + / + | | MG837363 | MG837430 | MG837296 | |
| *Epimedium chlorandrum* | LY01 | Leaf | Baoxing, Sichuan | + / + | + / + | + / + | | MG837364 | MG837431 | MG837297 | |
| *Epimedium chlorandrum* | LY02 | Leaf | Baoxing, Sichuan | + / + | + / + | + / + | | MG837365 | MG837432 | MG837298 | |
| *Epimedium chlorandrum* | LY03 | Leaf | Yaan, Sichuan | + / + | + / + | + / + | | MG837366 | MG837433 | MG837299 | |
| *Epimedium franchetii* | MYP01 | Leaf | Wufeng, Hubei | + / + | + / + | + / + | | MG837368 | MG837435 | MG837301 | |
| *Epimedium leptorrhizum** | QL01 | Leaf | Guiyang, Guizhou | + / + | + / + | + / - | | # | # | # | |
| *Epimedium leptorrhizum** | QL02 | Leaf | Guiyang, Guizhou | + / + | + / + | + / + | | MG837373 | MG837440 | MG837306 | |
| *Epimedium leptorrhizum** | QL03 | Leaf | Songtao, Guizhou | + / + | + / + | + / + | | MG837374 | MG837441 | MG837307 | |
| *Epimedium leptorrhizum** | QL04 | Leaf | Baojing, Guizhou | + / + | + / + | + / + | | MG837375 | MG837442 | MG837308 | |
| *Epimedium rhizomatosum* | QJ01 | Leaf | Mao, Sichuan | + / + | + / + | + / + | | MG837376 | MG837443 | MG837309 | |
| *Epimedium rhizomatosum* | QJ02 | Leaf | Mao, Sichuan | + / + | + / + | + / + | | MG837377 | MG837444 | MG837310 | |
| *Epimedium lishihchenii** | SZ01 | Leaf | Jiujiang, Jiangxi | + / + | + / + | + / + | | MG837383 | MG837450 | MG837316 | |
| *Epimedium sutchuenense** | SIC01 | Leaf | Zhenping, Shaanxi | + / + | + / + | + / + | | MG837386 | MG837453 | MG837319 | |
| *Epimedium glandulosopilosum* | XM01 | Leaf | Wuxi, Chongqing | + / + | + / + | + / + | | MG837396 | MG837463 | MG837329 | |
| *Epimedium ilicifolium* | ZP01 | Leaf | Zhenping, Shaanxi | + / + | + / + | + / + | | MG837402 | MG837469 | MG837335 | |
| *Epimedium zhushanense* | ZS01 | Leaf | Zhuxi, Hubei | + / + | + / + | + / + | | MG837405 | MG837472 | MG837338 | |
| *Epimedium zhushanense* | ZS02 | Leaf | Zhuxi, Hubei | + / + | + / + | + / + | | MG837406 | MG837473 | MG837339 | |
| *Epimedium dewuense** | DW01 | Leaf | Guiyang, Guizhou | + / + | + / + | + / + | | MG837354 | MG837421 | MG837287 | |
| *Epimedium dewuense** | DW02 | Leaf | Dejiang, Guizhou | + / + | + / + | + / + | | MG837355 | MG837422 | MG837288 | |
| *Epimedium sagittatum** | JY01 | Leaf | Jinzhai, Anhui | + / + | + / + | + / + | | MG837361 | MG837428 | MG837294 | |
| *Epimedium sagittatum** | JY02 | Leaf | Huangshan, Anhui | + / + | + / + | + / + | | MG837357 | MG837424 | MG837290 | |
| *Epimedium sagittatum** | JY03 | Leaf | Luotian, Hubei | + / + | + / + | + / + | | MG837358 | MG837425 | MG837291 | |
| *Epimedium sagittatum** | JY04 | Leaf | Jianghua, Hunan | + / + | + / + | + / + | | MG837359 | MG837426 | MG837292 | |
| *Epimedium sagittatum** | JY05 | Leaf | Jishou, Hunan | + / + | + / + | + / + | | MG837360 | MG837427 | MG837293 | |
| *Epimedium borealiguizhouense** | QB01 | Leaf | Guiyang, Guizhou | + / + | + / + | + / + | | MG837372 | MG837439 | MG837305 | |
| *Epimedium truncatum** | PX01 | Leaf | Zhangjiajie, Hunan | + / + | + / + | + / + | | MG837371 | MG837438 | MG837304 | |
| *Epimedium qingchengshanense* | QCS01 | Leaf | Dujiangyan, Sichuan | + / + | + / + | + / + | | MG837378 | MG837445 | MG837311 | |
| *Epimedium pubescens** | RM01 | Leaf | Longchang, Sichuan | + / + | + / + | + / + | | MG837379 | MG837446 | MG837312 | |
| *Epimedium pubescens** | RM02 | Leaf | Yaan, Sichuan | + / + | + / + | + / + | | MG837380 | MG837447 | MG837313 | |
| *Epimedium pubescens** | RM03 | Leaf | Dujiangyan, Sichuan | + / + | + / + | + / + | | MG837381 | MG837448 | MG837314 | |
| *Epimedium myrianthum** | TPS01 | Leaf | Kaiyang, Guizhou | + / + | + / + | + / + | | MG837388 | MG837455 | MG837321 | |
| *Epimedium myrianthum** | TPS02 | Leaf | Sansui, Guizhou | + / + | + / + | + / + | | MG837389 | MG837456 | MG837322 | |
| *Epimedium myrianthum** | TPS03 | Leaf | Yuping, Guizhou | + / + | + / + | + / + | | MG837390 | MG837457 | MG837323 | |
| *Epimedium myrianthum** | TPS04 | Leaf | Guiyang, Guizhou | + / + | + / + | + / + | | MG837391 | MG837458 | MG837324 | |
| *Epimedium brevicornu** | XY01 | Leaf | Hubei | + / + | + / + | + / + | | MG837397 | MG837464 | MG837330 | |
| *Epimedium brevicornu** | XY02 | Leaf | Hubei | + / + | + / + | + / + | | MH252070 | MH252073 | MH252067 | |
| *Epimedium stellulatum* | XH01 | Leaf | Nanzheng, Shaanxi | + / + | + / + | + / + | | MG837398 | MG837465 | MG837331 | |
| *Epimedium coactum** | ZM01 | Leaf | Guiyang, Guizhou | + / + | + / + | + / + | | MG837399 | MG837466 | MG837332 | |
| *Epimedium coactum** | ZM02 | Leaf | Jianhe, Guizhou | + / + | + / + | + / + | | MG837400 | MG837467 | MG837333 | |
| *Epimedium dolichostemon* | CR01 | Leaf | Jianshi, Hubei | + / + | + / + | + / + | | MG837401 | MG837468 | MG837334 | |
| *Epimedium reticulatum* | GY01 | Leaf | Jinyang, Sichuan | + / + | + / + | + / + | | MG837356 | MG837423 | MG837289 | |
| *Epimedium platypetalum* | MW01 | Leaf | Nanzheng, Shaanxi | + / + | + / + | + / + | | MG837367 | MG837434 | MG837300 | |
| *Epimedium koreanum** | CX01 | Leaf | Jilin | + / + | + / + | + / + | | MG837347 | MG837414 | MG837280 | |
| *Epimedium koreanum** | CX02 | Leaf | Jilin | + / + | + / + | + / + | | MG837348 | MG837415 | MG837281 | |
| *Epimedium tianmenshanensis* | TMS01 | Leaf | Zhangjiajie, Hunan | + / + | + / + | + / + | | MG837387 | MG837454 | MG837320 | |
| *Epimedium xichangense* | XC01 | Leaf | Xichang, Sichuan | + / + | + / + | + / + | | MG837395 | MG837462 | MG837328 | |
| *Epimedium pudingense** | PD01 | Leaf | Puding, Guizhou | + / + | + / + | + / - | | # | # | # | |

“+”, positive; “-”, negative; “#”, not submitted; “*”, medicinally used species.

**Table S2.** List of universal primers and reaction conditions for candidate barcodes used in this study.

| **Marker** | **Name of primers** | **Primer sequences 5′-3′** | **PCR reaction conditions** |
| --- | --- | --- | --- |
| ITS | 5a fwd | CCTTATCATTTAGAGGAAGGAG | 94℃ 5 min |
|  | 4 rev | TCCTCCGCTTATTGATATGC | 94℃ 1 min, 50℃ 1 min, 72℃ 1.5 min + 3 sec/cycle, 30 cycles |
|  |  |  | 72℃ 7 min |
| *rbcL* | 1f | ATGTCACCACAAACAGAAAC | 95℃ 2 min |
|  | 724r | TCGCATGTACCTGCAGTAGC | 94℃ 1 min, 55℃ 30 sec, 72℃ 1 min, 34 cycles |
|  |  |  | 72℃ 7 min |
| *psbA*-*trnH* | fwd PA | GTTATGCATGAACGTAATGCTC | 94℃ 5 min |
|  | rev TH | CGCGCATGGTGGATTCACAATCC | 94℃ 1 min, 55℃ 1 min, 72℃ 1.5 min, 30 cycles |

**Table S3.** Inter- and intraspecific genetic divergences of the four loci.

| **Model** |  |  | **K2P distance** | | | |
| --- | --- | --- | --- | --- | --- | --- |
| **DNA barcode** | |  | *psbA*-*trnH* | ITS2 | ITS | *rbcL* |
| All inter-specific distance | |  | 0.0108±0.0036 | 0.0059±0.0059 | 0.0032±0.0027 | 0.0014±0.0011 |
| Theta prime | |  | 0.0107±0.0003 | 0.0050±0.0033 | 0.0027±0.0020 | 0.0015±0.0004 |
| Minimum interspecific distance | |  | 0.0039±0.0028 | 0 | 0 | 0 |
| All intraspecific distance | |  | 0.0095±0.0053 | 0.0058±0.0064 | 0.0030±0.0031 | 0.0016±0.0012 |
| Theta |  |  | 0.0106±0.0039 | 0.0052±0.0053 | 0.0029±0.0030 | 0.0016±0.0009 |
| Coalescent depth | |  | 0.0156±0.0050 | 0.0109±0.0104 | 0.0057±0.0052 | 0.0029±0.0014 |
