# Supplementary figures and images for: A Systematic Study on DNA Barcoding of Medicinally Important Genus Epimedium L. (Berberidaceae)
Source: Genes (Basel). 2018 Dec 17;9(12):637. doi: 10.3390/genes9120637 (PMC6316794; doi:10.3390/genes9120637)

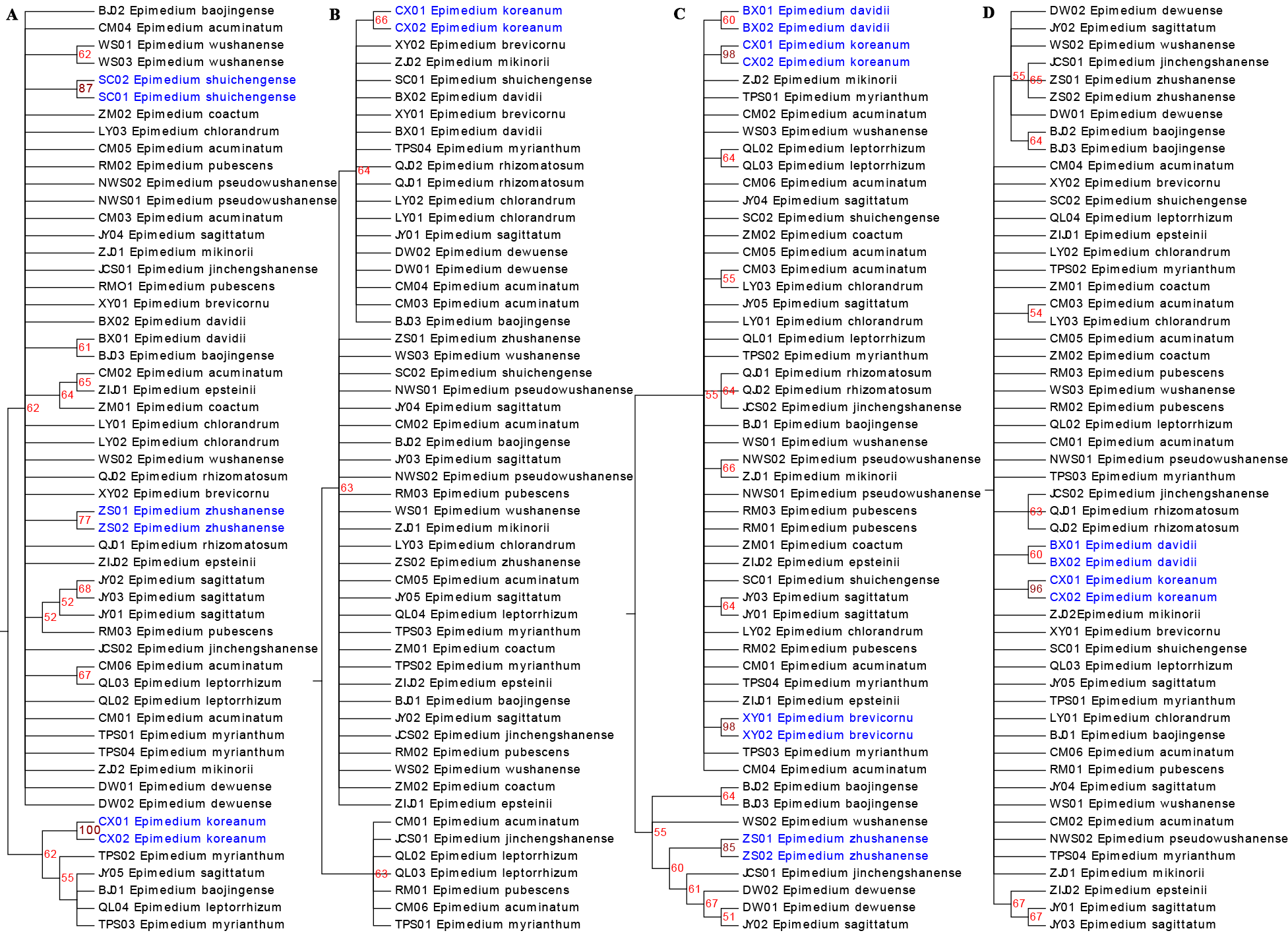

Supplement: Supplementary file 1 [file genes-09-00637-s001.zip › Figure S1.tif]

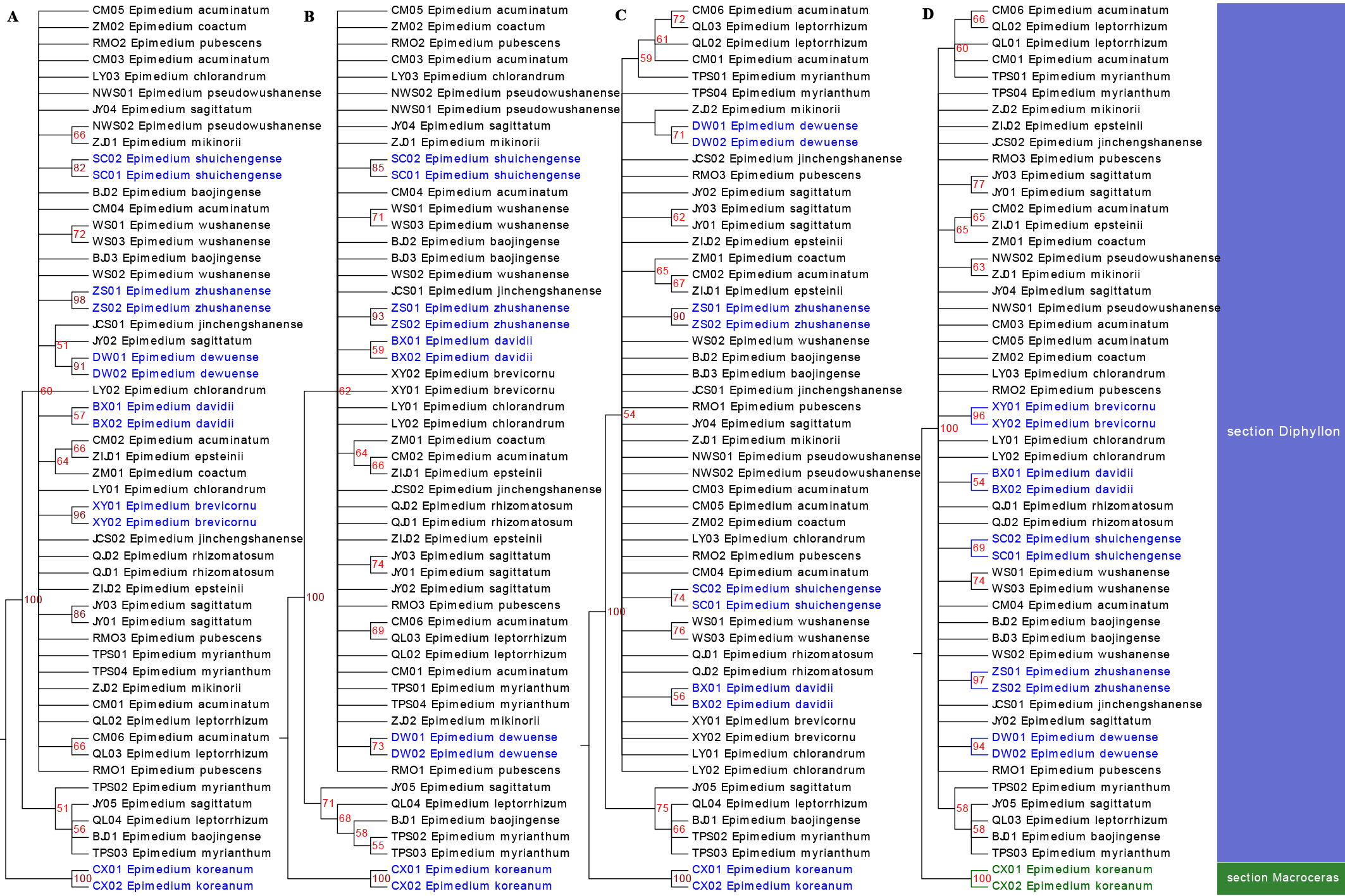

Supplement: Supplementary file 1 [file genes-09-00637-s001.zip › Figure S2.tif]
